# Child growth in urban deprived settings: Does household poverty status matter? At which stage of child development?

**DOI:** 10.1016/j.healthplace.2011.12.003

**Published:** 2012-03

**Authors:** Jean Christophe Fotso, Nyovani Madise, Angela Baschieri, John Cleland, Eliya Zulu, Martin Kavao Mutua, Hildah Essendi

**Affiliations:** a African Population and Health Research Center (APHRC), Population Dynamics and Reproductive Health, APHRC Campus, Kirawa Road, Off Peponi Road, P.O. Box 10787, 00100 GPO, Nairobi, Kenya; b School of Social Sciences, University of Southampton**,** Southampton, UK; c Centre for Population Studies, London School of Hygiene & Tropical Medicine (LSHTM), London, UK; d African Institute for Development Policy (AFIDEP), Nairobi, Kenya; e African Population and Health Research Center (APHRC), Nairobi, Kenya

**Keywords:** Child undernutrition, Urban poverty, Kenya, Longitudinal study, Demographic surveillance system

## Abstract

This paper uses longitudinal data from two informal settlements of Nairobi, Kenya to examine patterns of child growth and how these are affected by four different dimensions of poverty at the household level namely, expenditures poverty, assets poverty, food poverty, and subjective poverty. The descriptive results show a grim picture, with the prevalence of overall stunting reaching nearly 60% in the age group 15–17 months and remaining almost constant thereafter. There is a strong association between food poverty and stunting among children aged 6–11 months (*p*<0.01), while assets poverty and subjective poverty have stronger relationships (*p*<0.01) with undernutrition at older age (24 months or older for assets poverty, and 12 months or older for subjective poverty). The effect of expenditures poverty does not reach statistical significant in any age group. These findings shed light on the degree of vulnerability of urban poor infants and children and on the influences of various aspects of poverty measures.

## Highlights

► We assess how infant and child undernutrition varies across four dimensions of poverty status. ► There is a strong association between food poverty and stunting among children aged 6–11 months. ► Assets poverty and subjective poverty have stronger effects on undernutrition at older age. ► The effect of expenditures poverty does not reach statistical significant in any age group. ► These findings suggest the need to design and scale up nutrition interventions among the urban poor.

## 1 Introduction

Approximately 167 million children under five years of age – almost one-third of the developing world's children – are undernourished, a condition that contributes to almost 60% of all child deaths in developing countries (Levinston and Bassett, 2007; FAO, 2005; Pelletier et al., 1995). The greatest burden of undernutrition is often among the poorest households who are more likely to experience food and nutritional insecurity as a result of lack of resources and food, low levels of education and nutritional health information, and poor access to and utilization of healthcare (World Bank, 2010; Peña and Bacallao, 2002; FAO, 1997). Many studies assessing the impact of poverty on malnutrition are limited by lack of detailed time series data that would demonstrate how both short term and long term changes in wellbeing impact child growth.

Many datasets that are used to analyze malnutrition often have deficient measures of poverty and, therefore, fail to tease out how various dimensions of poverty affect child health at different stages of child growth (Falkingham and Namazie, 2002). Furthermore, while it is generally understood that poverty plays a big role in affecting child malnutrition, few studies have examined how varying levels and dimensions of poverty in poor and uniquely vulnerable populations like urban slum settlements affect child malnutrition. This study contributes to filling these knowledge gaps by using uniquely rich longitudinal data collected from two informal settlements in Nairobi City in order to examine patterns of child growth and how these are affected by four different dimensions of poverty, and at different stages of child development.

### 1.1 Infant and child undernutrition and the millennium development goals

Poor nutrition hinders progress towards most of the millennium development goals (MDGs) as its impacts from infancy can be felt throughout the lifecycle. Undernutrition results in increased health care cost and low productivity, thereby perpetuating the poverty cycle and slowing progress towards MDG 1. Many children die from treatable infectious diseases including diarrhea, pneumonia, malaria, and measles due to weakened immune systems arising from undernutrition – MDG 4. Undernourishment impairs children's learning abilities and cognitive development, and the poor health arising from malnourishment may lead to delayed enrollment in school, high school drop-out rates, and poor educational attainments – MDG 2 (FAO, 2005; ACC/SCN, 2004; Amuyunzu-Nyamongo and Taffa, 2004; Frongillo et al., 1997).

Indeed, a number of studies have shown that early childhood health influences the achievement of traits that are rewarded in the labor market such as improved cognitive performance, higher educational attainment, and positive personality attributes (Palloni, 2006). In addition, evidence exists of the importance of investment in nutrition for positive economic performance (World Bank, 2010; Fogel, 1994; Strauss, 1986). Furthermore, female children who are undernourished are later more likely to give birth to low birth weight children and to experience maternal mortality (Peña and Bacallao, 2002; Pelletier et al., 1995; UNICEF, 1990).

Routine measurements of weight and height are internationally accepted standards of monitoring nutritional status and growth in children (FAO, 1997; WHO, 1995). Child growth monitoring is largely driven by the evidence that if malnutrition in children is not addressed before two years of age, there may be irreversible damages on the child's intellectual development (Victora et al., 2010; World Bank, 2010; Levinston and Bassett, 2007). However, many studies looking at child nutritional status use cross-sectional data which does not allow for proper follow-up of changes in individual children' growth trajectories. Longitudinal studies are more powerful in monitoring child growth in an age-based perspective, and for providing robust evidence upon which to inform policies and the design of effective interventions. Methodologically, a prospective study design is immensely more powerful – than cross-sectional data – as it links a sequence of events to the life-course of a particular individual, with ability to make cause inference enhanced by temporal ordering. Indeed longitudinal data are more suitable to measure within-individual change, allowing for the possibility of controlling for unobserved individual characteristics (Arjas and Parner, 2004; Singer and Willett, 2003).

### 1.2 Poverty and undernutrition among the urban poor

In this article, we focus on the urban poor who, until recently, have not received much attention from scholars. High population growth in sub-Saharan African cities over the last few decades, fuelled by both high natural increase and rural-to-urban migration, has contributed to growing urban poverty since the pace of population increase has overwhelmed the ability of weak economies and local authorities to generate employment and provide sufficient affordable housing and infrastructure (Cohen, 2004). Consequently, a large proportion of urban dwellers in Africa are now living in unplanned settlements within or on the periphery of large cities where poverty levels are high (Brockerhoff, 2000; Montgomery and Hewett, 2004). Urban slums are characterized by overcrowding, poor ventilation, lack of portable water, dilapidated housing, high levels of criminal activity, constant flow of immigrants and rapid spread of diseases. These conditions expose young children to health hazards and heightened risk of morbidity and mortality (APHRC, 2002). The effect of urban poverty on child health cannot be overemphasized as data show that in some countries such as Kenya and Zambia, children in informal urban settlements have poorer health outcomes than rural children (Fotso et al., 2007). Under-five mortality in Nairobi slums for instance, is at 151 per thousand births compared to 62 in Nairobi as a whole and 113 in rural Kenya (APHRC, 2002).

Many studies on child undernutrition have neglected the urban poor and particularly the slum populations. Child undernutrition, like many other child health indicators, is expected to be poor among this group since poverty, coupled with environmental hazards, is likely to produce synergistic hazardous effects on children. Understanding the dynamics of child growth in such populations is therefore important for child survival strategies. Though their environment is similar, slum populations vary in the nature and degree of deprivation, as highlighted by Zulu et al. (2011). For instance, poverty levels vary widely across different slums, between male and female-headed households, and by duration of stay in slum settlements. Poverty incidence across 14 villages in two slums of Nairobi, Kenya (Korogocho and Viwandani) varied from 42% to 78%. Furthermore, the wealthiest decile in either of the two slums had expenditures (per adult equivalent) 14 times higher than the bottom in 2006 (World Bank, 2008).

### 1.3 Conceptualization and measurement of poverty status

Over the last two decades there has been widespread acceptance of the view that poverty is more than a lack of material resources (Mitlin, 2003; Falkingham and Namazie, 2002). The United Nations' Copenhagen summit in 1995 set out a general definition of poverty that recognised that human development goes beyond purely economic factors. Absolute poverty was defined as “a condition characterised by severe deprivation of basic human needs including food, safe drinking water, sanitation facilities, health, shelter, education and information” (UN, 1995, para 19, Chapter 2). These common understandings led to the development of a range of poverty measures aimed at capturing the multidimensionality of poverty (Bourguignon and Chakravarty, 2003).

Household poverty measures based on household income or expenditures are related to current employment circumstances and, in the case of urban deprived settings, on erratic income opportunities provided by the informal labour markets. In the face of perceived transitory shocks, households may reduce their consumption of food or household expenditures on other items, while in the face of more recurrent shocks, households might sell off their possessions to sustain their level of consumption or expenditures (Atkinson, 1987). Expenditure-related poverty is likely to provide some insights into the current ability to cater for households' basic food needs. Its effect on child health is expected to vary with child's age, with a stronger effect on children who have completed the weaning period. Poverty measures based on household ownership of assets represent the long-run accumulation of household wealth (Filmer and Pritchett, 2001; Montgomery et al., 2000), and several studies have reported strong association with child health outcomes (Gwatkin et al., 2000; Fotso and Kuate-Defo, 2005).

Alternative measures proposed in the literature are subjective measures of wellbeing derived by asking the head of the household to rank the household welfare status (Pradhan and Ravallion, 2000). Subjective measures of wellbeing have been reported to be associated with various markers of socio-economic status and are thought to better reflect the household's social position by taking into account past and future prospects. Some studies have found that such subjective measures are strongly associated with health outcomes (Manoux et al., 2005). Food security measures have also been developed by asking respondents to describe the food availability in the household and whether the household experienced periods of food shortages (Keenan et al. 2001; Frongillo, 1999). These measures were developed partly to gain an understanding of household food availability and the intra-household resources allocation (specifically how food purchases are allocated amongst the members of the household), and are likely to provide better understanding of the relationship between food shortages and child's nutrition, especially among older children.

## 2 Data and methods

### 2.1 Study setting

The study settings are two informal settlements of Nairobi, Kenya, namely, Viwandani and Korogocho where the African Population and Health Research Centre (APHRC) runs a longitudinal demographic surveillance system referred to as the Nairobi Urban Health and Demographic Surveillance System (NUHDSS). The NUHDSS has been in operation since 2002 and has about 60,000 registered inhabitants in nearly 20,000 households. These two densely populated communities have higher unemployment, poverty, crime, poor sanitation and generally poorer health indicators than Nairobi as a whole (APHRC, 2002). The two communities, however, exhibit structural differences: Viwandani is bordered by an industrial area and attracts relatively younger, more educated, and shorter term migrants, while the population in Korogocho is more stable and has higher levels of co-residence of spouses (Emina et al., 2011).

### 2.2 Data

This study uses data from the Maternal and Child Health (MCH) component of a broader project entitled “Urbanization, Poverty and Health Dynamics” being implemented in the NUHDSS. All NUHDSS female members who gave birth since September 2006 and their children were enrolled in the project, and anthropometric measurements taken. Updates were done during follow-up visits every four months, and also when new children were recruited into the study for the first time to form new cohorts. Some children could not be immediately traced until after several visits due to the high population mobility in urban poor settings. For the purpose of this study, we use data on 3693 children from six cohorts as described in Table 1. These children contribute data at different time-points (surveys) totaling 14,410 observations. The first baseline observations (Cohort 1 and survey 1) took place between February and April 2007 with follow-up visits and new recruitments made routinely thereafter. The first wave of cohort 3 was done during a prolonged period (between October 2007 and May 2008) as a result of the political and social instability that followed Kenya's 2007 elections.

The data in Table 1 show a relatively high level of attrition across successive waves. For instance, of the 568 children enrolled in the first cohort, 474 were successfully re-contacted in the first follow-up, and only 178 were reached during the eighth visit, for an average annual attrition rate of about 24%. The average annual attrition rate for the other cohorts ranged from 21 to 28%. Once a year the NUHDSS collects data on various aspects of well-being at the household level. The questionnaires cover monthly expenditures (on rent, food, energy, water, transport, electricity, health care, and school fees), assets (or possessions), dwelling characteristics (floor, wall, roof, drinking water, toilets, and garbage collection), subjective poverty on a scale from 1 (poorest) to 10 (richest), and access to food (e.g. number and quantity of meals, failure to eat, going to bed hungry). These data for 2007, 2008 and 2009 are also used in the analysis.

### 2.3 Dependent variables

The dependent variable is based on height-for-age *Z*-scores (HAZ), computed using the 2000 CDC growth reference standards using zanthro command in STATA. While child weight-for-age fluctuates with environmental influences such as acute infections and poor nutritional intake, the height-for-age indicator represents a long term measure of health or chronic undernourishment (FAO, 1997). As recommended by the World Health Organization, overall stunting is defined as HAZ below −2 standard deviations (SD) from the median of the WHO/NCHS reference, while severe stunting is defined as HAZ below −3SD from the median of the WHO/NCHS reference (WHO, 1995, 2010). HAZ score below −2SD for children in the age group below 2–3 years represents stunted growth which reflects a continuing process of ‘failing to grow’ or chronic malnutrition. In a healthy, well-nourished population of children, it is expected that approximately 2.3% of children will fall below two standard deviations of the reference population and will be classified as stunted, wasted or underweight (WHO, 1995). The World Health Organization considers the severity of malnutrition to be ‘high’ when the prevalence of stunting exceeds 30% and wasting reaches 10%. High levels of stunted growth are often associated with poor socio-economic conditions, frequent illness and inappropriate healthcare practices (WHO, 1995). In this and other similar studies, infants were measured in the recumbent position and ‘length’ was used rather than ‘height’.

### 2.4 Key predictors

We operationalize alternative measures of poverty which capture not only the money-metric dimension, but also the broader aspects of human deprivation. First, we constructed a money-metric indicator of poverty using information on monthly household consumption. This indicator allows us to assess the relationship between access to cash income and child growth. Second, we derived an assets index using information on household ownership of durable assets. As indicated earlier, the assets index is considered a good measure of long-term wealth, and is expected to have an impact on stunting which represents a long term nutritional deficiency. Third, we derived a food poverty index using information of household's access to food. This index allows us to assess the effect of household food insecurity on child growth. Lastly, we included a measure of subjective poverty, derived from households' perceptions of their relative wealth status in the community, on a scale from 1 (poorest) to 10 (richest). Table 2 describes the five alternative measures of household welfare.

Apart from the subjective poverty variable which was recoded in three categories using the cut-off points of three and six, the three other welfare indicators were recoded as *tertiles.* The categories were labeled “poorest”, “middle” and “least poor”. All four measures of poverty are time varying: the 2007 poverty indices were linked to the 2007 anthropometric data, the same for the 2008 and 2009 data. All poverty variables were measured at the NUHDSS level and tertiles derived after merging with the MCH data. There was about eight percent of missing values due to the fact that not all households had poverty information for the three time points. These missing values were imputed using the STATA add-on for imputation by chained equations (ICE) procedures (Royston, 2005). The following variables were used in the imputation equations: village where the household is located; mother's marital status, age, education and parity at the time of the first interview; household size; slum of residence; as well as the values of poverty measures for the preceding and/or the following time point. There were 100 observations with missing welfare data that were excluded from the analyses, hence a final sample of 14,310 observations from 3692 children.

### 2.5 Control variables

In the models we control for a set of characteristics and the child, mother, household and community levels which previous studies have hypothesized to have an impact on child growth. These include the sex and age of the child, and child's mother's education, length of stay in the study area, marital status, and parity. Besides mother's parity, we also control for household size since children may not necessary live with their biological parents. Using PCA, we also construct a household environment index from information on the type of dwelling's floor, wall and roof; toilet facilities, the type of drinking water source and garbage collection – factors expected to have a direct effect on risk of infections. Finally, we control for the slum of residence (Korogocho or Viwandani).

### 2.6 Methods of analysis

The analysis is conducted in three steps: First, univariate and bivariate analyses are used to describe the patterns of stunting as the children age, and to depict the differences across the five poverty measures. Second, four multivariate models are used to test robustness of each poverty measure as a predictor of child growth and development and the statistical significance of the differences observed in the descriptive phase. Third, we stratify the analysis by age to examine how the overall effect of poverty on child nutritional status may vary by age. Given that the data are made up of repeated longitudinal observations, we use the random intercept multilevel models to control for clustering of observations at child level. The model is specified as follows:

(1) $$\{\begin{array}{l} Logit(\pi_{ij})=\ln\left[\frac{\pi_{ij}}{1-\pi_{ij}}\right]=\beta_{0j}+\sum_{k=1}^{p}\beta_{k}x_{ij}^{\left(k\right)}\\ \beta_{0j}=\beta_{0}+u_{0j} \end{array}$$

where *i* and *j* refer to the observation and child, respectively; *π*_*ij*_ is the probability that the child referenced (*i*, *j*) is stunted; $x_{ij}^{\left(k\right)}$ is the *k*th covariate; *β*_0*j*_ represents the intercept modelled to randomly vary between children; *β*_*k*_ is the regression coefficients of the *k*th explanatory variables; and *u*_0*j*_ is the random coefficient distributed as $N(0,\sigma_{u}^{2})$ (Rasbash et al., 2002). The equations used to fit the interaction models are derived from eq. (1). Models are fitted using the STATA “*xtlogit*” command. The third category (least poor) is used as the reference group for all five measures of poverty. The presentation of results will focus primarily on the coefficient of the first category (poorest).

## 3 Results

### 3.1 Descriptive analysis

#### 3.1.1 Sample characteristics

Table 3a shows that the four measures of welfare are weakly correlated, suggesting that they indeed capture different aspects of well-being. The correlation coefficients range from as low as 0.07 (between expenditures poverty and subjective poverty) to 0.31 (between expenditures poverty and food poverty), with intermediary values around 0.25 (between food poverty and assets poverty, and between food poverty and subjective poverty). As an illustration of the weak correlation, among observations that are classified poorest (lowest tertile) based on the expenditures poverty measure, only 42% are also classified as poorest based on the assets poverty variable, while 33% and 25% are classified as middle (second tertile) and least poor (highest tertile), respectively. The analysis further reveals that the correlation between the four measures is mainly noticeable in Viwandani, except for the relationship between subjective poverty and assets poverty which is stronger in Korogocho (see Appendix 1).

Tables 3b and 3c show the characteristics of children included in the analysis at enrollment and from the full sample (observations). As can be seen, the distribution of the money-metric, assets and food poverty variables is in line with the coding of the variables as tertiles. As for subjective poverty, about 47% of observations were from households that were self-ranked 4 or 5 on the 10-point scale of subjective poverty. A high proportion of children enrolled in the study were from mothers who had completed primary education (46.5%), had spent two years or less in the study area (44.9%), were in a union (85.0%), or had parity of three or higher (39.1%). More than 60% of the children enrolled were from households with four or more members. Finally, as is the case in the NUHDSS at large, a slightly larger proportion of observations (about 51.1%) were from Korogocho.

#### 3.1.2 Child growth and bivariate analysis of the influence of poverty

**Fig. 1:**
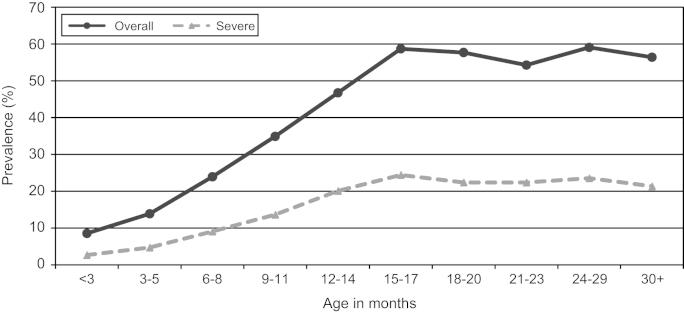
Prevalence of stunting.

Fig. 1 shows a grim picture with the prevalence of overall stunting increasing sharply from less than 9% from the first three months of life to nearly 60% in the age group 15–17 months, and remaining almost constant thereafter. More worrying is the fact that severe stunting (life-threatening cases) rises from less than 3% during the first three months of life to about 25% among children in the 15–17 month age group.

Table 4 illustrates the bivariate association between the four dimensions of poverty and stunted growth based on the overall sample, and stratified by child age (<6, 6–11, 12–23 and 24+). The association between e*xpenditures poverty* and stunting appears in the opposite direction in the overall sample; it is strong and in the expected direction among children aged 12–23 months (*p*<0.01), and more so among children aged 24 months or older (*p*<0.05). For example, children aged 24 months or older from the poorest households are 3.1 times as likely to be stunted, compared to their counterparts of the same age group from the least poor households. *Food poverty* and *assets poverty* exhibit a strong relationship with child undernutrition. The associations are statistically significant in the overall sample and in all age groups, except among infants aged less than six months, which is not surprising since many children are likely to be breastfed during the first six months of life. Noticeably, among children aged 24 months or older, those from the poorest households with regard to food poverty, are nearly 3.8 times as likely as their counterparts from the least poor households (*p*<0.10). The gap is substantially wider with regard to assets poverty (odds ratio of about 8). Finally, the bivariate relationship of subjective poverty and stunted growth is apparent in the overall sample (*p*<0.01) and among children aged 12–23 months (*p*<0.05), and is markedly strong among older children with an odds ratio of 12.4 (*p*<0.01).

#### 3.1.3 Multivariate analysis of poverty and child growth: what is the age pattern of the relationships?

The results of the multivariate analysis on the influences of poverty on child undernutrition in the overall sample and by age group, are summarized in Table 5. Among all children, assets poverty and subjective poverty are significantly associated with stunted growth (*p*<0.05), while the relationships between expenditures poverty and food security and stunting are in the expected direction but fails to reach statistical significance at the level of 0.10.

The stratification by age shows a distinctive pattern of relationship between the welfare variables and child growth. There is a strong association between the food poverty measure and child stunting among children aged 6–11 months (*p*<0.01). This result suggests that household food insecurity has a strong effect on children at young age, with poorest children aged 6–11 months being 2.2 times more likely to experience stunted growth than children in the same age group from richer households. On the other hand, the assets poverty measure, which represents long-term household wealth, has a stronger association at older age (24 months or older), with children from poorest households nearly 3.9 times more likely to be stunted, compared with the ones from the least poor households. The effect of subjective poverty is statistically significant among children in the 12–23 months age group (*p*<0.05), and is very strong among older children (*p*<0.01), with an odds ratio of 4.4. The association of expenditures poverty with stunting is not apparent in the overall sample or in the results by age.

#### 3.1.4 Other determinants of child undernutrition

The full results of the multivariate analyses are reported in Table 6. The associations between stunting and each of the nine control variables do not change much across the four models. Consistent with the age pattern depicted in Fig. 1, the multivariate results show a significantly large difference in the probability of being stunted for children aged 24 months or older compared to children less than a year (*p*<0.001 across all five models). Our results show that across all five models, boys are significantly worse-off than girls, being about 2.5 times more likely to be stunted (*p*<0.001). As we expected, children born to mothers with secondary or higher education are significantly better-off, compared with those born to mothers who had no education or had not completed primary education (*p*<0.01 in all five models). Children born to mothers who had completed primary education tended to be better-off compared with their counterparts born to mothers with no education or incomplete primary education, but the difference did not reach statistical significance.

Being in a union appears to be a protective factor since children born to mothers who are not in a union were about 35% more likely to be stunted, compared to those born to mothers who are in a union (*p*<0.001 across all five models). As expected, the likelihood of stunting in children increases significantly with mothers' parity (*p*<0.01 or *p*<0.05 in all five models). There was no association between household size or length of stay in the study area, and child stunting. Finally, while the household environment index does not emerge as a predictor of child stunting, children living in Viwandani tended to be better-off than their counterparts from Korogocho, with the difference reaching statistical significance in the models with expenditures poverty and subjective poverty (*p*<0.10).

## 4 Discussion and conclusion

### 4.1 Magnitude of child undernutrition among the urban poor

The descriptive results show a sharp increase in the prevalence of child stunting from about 10% in early infancy to about 60% by the age of 15 months, suggesting that addressing children's nutritional status should be prioritized by programs aimed at improving child health and survival among the urban poor. Our finding points to huge poor/rich gaps in nutritional status in urban Kenya since national data from the 2008/09 Kenya Demographic and Health Survey indicates that the prevalence of stunted growth among children aged 15 months or older stands at around 27% in urban Kenya and about 45% in rural Kenya, far lower than the prevalence of 60% recorded in the slums of Nairobi for this age group. Our results are also in line with other studies that have reported the unique vulnerability of the urban poor compared to other urban as well as rural residents (Fotso, 2006; Menon et al., 2000; APHRC, 2002). A study by Kennedy et al. (2006) which compared nutritional status of urban and rural children in Angola, Central African Republic, and Senegal found that once wealth was controlled for, urban and rural children had the same likelihood of being stunted, leading further support to the view that poverty is a key predictor of malnutrition in Africa, and other developing countries.

The poor nutritional status of children in this community is to be expected. The study participants live in overcrowded make-shift settlements characterized by poor environment and housing, and poor access to safe water, food and health services (Zulu et al. 2011; APHRC, 2002; Zulu et al., 2002). Previous research conducted in the same community shows that compared with children from other parts of Kenya, including the larger city of Nairobi, those living in these slums have a lower likelihood of being vaccinated and a higher infant mortality rates, and their mothers have higher maternal mortality (APHRC 2002). Overall, our findings provide further evidence to the concept of “the urban health penalty” which posits that cities concentrate poor people in defined geographic areas and expose residents of these areas to unhealthy environments that result in a disproportionate burden of poor health (Goebel et al., 2010; Fotso, 2007).

### 4.2 How alternative poverty measures affect child malnutrition in the context of urban poverty?

Results of the multivariate models reveal that the effect of household welfare on child stunting depends on the measure of welfare chosen. In addition, the results from the analysis stratified by age also reveal that different aspects of household welfare affect growth of children differently at different points in the child growth cycle. The relationship between **food poverty** and child growth should be considered a dynamic experience, varying through graded levels of severity ranging from uncertainty and anxiety about food to the extreme case of hunger. In addition, while food insecurity and hunger may lead to malnutrition over time, they may occur without the overt signs of sub-optimal nutritional status (Maxwell, 2001). Our results show that household food poverty is a strong determinant of child growth. The strongest effect appears for children between 6 and 11 months, suggesting that household food insecurity particularly affects children's nutritional status around the period when complementary feeding is introduced. Few studies have looked explicitly at the relationship between food security and nutritional status. One such study used data from the Kailali district in Nepal and found no association between food security and stunting among children aged 6–23 months (Osei et al., 2010).

While greater income or consumption at the household level is generally associated with greater investment in food products, better hygiene, access to clean water and better care of children's nutritional status by mothers (Haddad et al., 1999), the multivariate results of this study did not find a consistent association between **money-metric measure** of household welfare and child stunting. This finding is surprising as this measure is often regarded as a more superior indicator of poverty compared to the other measures used in the study. Furthermore, in urban settings where the majority of households buy their own food, lack of income has been reported to be the main cause of food insecurity (Menon et al., 2000; Haddad et al., 1999). A study in Accra (Ghana) found that households purchase 90% of their food (Maxwell et al., 2000). Urban dwellers, unlike their rural counterparts cannot rely on their own production for food, and food expenditures can make up a large percentage of total household expenditures (42% in Korogocho and 35% in Viwandani). The absence of a relationship may reflect frequent fluctuations in cash income (and thus expenditures) in a setting where few have regular jobs, or suggest that the main effect of living in a better off household (as represented by the money-metric indicator) operates via the better ability of the household to provide better hygiene and better living conditions for children.

From this study, **assets poverty** emerged as a strong predictor of child stunting, with poor households being 38% more likely to have a child stunted than the richest. This association also varies by age and older children living in the poorest households are 3.9 times more likely to be stunted than children living in the richest household. These findings confirmed the importance of long term household welfare on child growth found in other studies (Filmer and Pritchett, 2001; Sahn and Alderman, 1997). The effect of **subjective poverty index** is also particularly strong for children two years and older. Older children living in households that rate themselves as poorest are 4.4 times as likely to be stunted as children living in households that do not consider themselves poor. The stronger effect of subjective poverty on stunting at older ages confirms further that child stunting is the cumulative effect of long term deprivation, and not necessarily that this effect is only operating at this specific age group.

Noticeably, the effects of the poverty measures on child undernutrition vary greatly by slum of residence as shown in Appendix 2. Among all children, assets poverty is a strong predictor of child health in Korogocho, while the three others are at play in Viwandani.

### 4.3 Other determinants of child malnutrition

As shown in the descriptive and the multivariate models, the single most important determinant of stunting is age, a result in agreement with other studies (Victora et al., 2010; Adair and Guilkey, 1997). This relationship demonstrates the disadvantage of children born in poor countries where the environment and poverty combine to progressively affect health and wellbeing. Male children from the study area are 2.5 times more likely to be stunted than girls. This finding on the vulnerability of boys in terms of nutritional status has also been well documented (Marcoux, 2002). Adair and Guilkey (1997) reported that Filipino males were more likely to become stunted in the first year of life, whereas females were more likely to become stunted in the second year of life. The gender difference in nutritional status is often attributed to the physiological disadvantage of boys at birth and during infancy (Marcoux, 2002), with speculation that the absence of this inherent male disadvantage (for example in India and China), could be attributed to infant rearing practices that favour the male child (Griffiths et al., 2004).

The relationship between mother's education and child malnutrition in this study replicates well established findings documented in other studies and other contexts (De Silva and Harpham, 2007; Fotso and Kuate-Defo, 2005; Madise and Mpoma, 1997; Cleland and Van Ginneken, 1988). In contrast to these studies, however, our results show that completed primary education does not confer any child health advantage. However, mothers with secondary or higher schooling are clearly able to transcend conditions of poverty and poor environment in matters of child nutrition and health care. Marital status and parity all have substantial effects on child health and in the expected direction.

In contrast to a study from the same study communities that showed a negative impact on the duration of stay on child mortality (Konseiga et al., 2009), our results show that mother's length of stay in the slums is not significantly associated with child stunting. As shown in other studies (Fotso and Kuate-Defo, 2005), household size is not related with child nutrition and overall health after controlling for socio-economic status. However, in their national level study in six African countries, Madise et al. (1999) reported that the number of under-five children in a household has a negative influence on weight-for-age *z*-scores of children aged 1–35 months. There is no significant association between household environment index and child growth, implying that, in this population, water supply, sanitation and other components of household living conditions do not affect children's growth patterns. This unexpected result might be due to the fact that variation in household amenities is narrow and/or that environmental factors outside the household dominate any effect of this index.

Expectedly, Viwandani children are significantly better-off than their counterparts from Korogocho. This difference by slum of residence agrees with findings from other work conducted in the study area (Emina et al., 2011; Zulu et al., 2011).

### 4.4 Limitations of the study

There are a few caveats to the findings from this study. First, due to high population mobility in the informal settlements, the longitudinal data used in the study recorded a high attrition as can be seen in Table 2. Second, there was about 15% of missing values for most of the poverty measures at the household level. Linear interpolation was used to reduce the number of missing cases below 5%, but household with no poverty data in the three years covered by the data were excluded from the analyses. Third and finally, we recognize the difficulty to accurately measure household expenditures in the context of developing countries. Also, our data do not have information on the socioeconomic status (e.g. education and working status) of mothers' partners – for those in union – which may have been important confounders.

Despite these limitations, our findings shed light on the degree of vulnerability of infants and children in the slums of Nairobi, Kenya, and on the influences of various aspects of poverty measures. They suggest the need to design and scale up nutrition interventions including growth monitoring and promotion, and appropriate feeding practices (Victora et al., 2010; Levinston and Bassett, 2007). Attention to urban areas is warranted given the forecast that by 2030, the majority of sub-Saharan Africa's population will be living in urban areas (UN, 2009; Harpham, 2009).

## Figures and Tables

**Table A1:** Correlation between four measures of poverty, stratified by slum of residence.

	Expenditures poverty	Assets poverty	Food poverty	Subjective poverty
**In Korogocho**				
Expenditures poverty	1.000			
Assets poverty	−0.010 (0.375)	1.000		
Food poverty	0.087 (0.000)	−0.077 (0.000)	1.000	
Subjective poverty	−0.059 (0.000)	0.126 (0.000)	0.090 (0.000)	1.000

**In Viwandani**				
Expenditures poverty	1.000			
Assets poverty	0.172 (0.000)	1.000		
Food poverty	0.237 (0.000)	0.242 (0.000)	1.000	
Subjective poverty	0.050 (0.000)	0.053 (0.000)	0.211 (0.000)	1.000

**Table A2:** Bivariate and multivariate analyses of the effects of household poverty status on child stunting, stratified by slum of residence.

	Poverty measure			
	Model with expenditures poverty	Model with assets poverty	Model with food poverty	Model with subjective poverty
**Bivariate analysis**				
Korogocho				
Poorest	0.75⁎⁎	1.33⁎	1.17	1.33⁎⁎
Middle	0.81⁎	1.15	0.91	1.47⁎⁎⁎
Least poor	1.00	1.00	1.00	1.00

Viwandani				
Poorest	0.87⁎⁎	0.92	1.32⁎	1.20†
Middle	0.88⁎	0.86	1.21⁎⁎	1.11
Least poor	1.00	1.00	1.00	1.00

**Multivariate analysis**^a^				
Korogocho				
Poorest	0.97	1.60⁎⁎⁎	0.94	1.21
Middle	0.93	1.23†	1.01	1.27⁎
Least poor	1.00	1.00	1.00	1.00

Viwandani				
Poorest	1.30†	1.05	1.29†	1.44⁎
Middle	1.10	0.90	1.32⁎	1.03
Least poor	1.00	1.00	1.00	1.00

a Analyses include all control variables as in Table 6.

† *p*<.10.

⁎ *p*<.05.

⁎⁎ *p*<.01.

⁎⁎⁎ *p*<.001.

**Table 1:** Sample size.

Survey period ==>	Survey 1	Survey 2	Survey 3	Survey 4	Survey 5	Survey 6	Survey 7	Survey 8	Total number of observa-tions	Average annual attrition (%)^a^	Duration		Rate of attrition	
	Feb–Apr 2007	Jul–Aug 2007	Oct–Dec 2007&-Mar–Apr 2008	May–Aug 2008	Sep 2008–Jan 2009	Feb–May 2009	Jun–Sep 2009	Oct–Jan 2010						
Cohort 1	**568**	474	350	311	269	228	198	178	2576	24.2	34	2.8	−0.336	−24.2
Cohort 2		**416**	334	295	268	235	216	196	1960	21.2	30	2.5	−0.260	−21.2
Cohort 3			**831**	663	583	526	479	438	3520	21.8	26	2.2	−0.256	−21.8
Cohort 4				**778**	713	648	558	448	3145	28.3	18	1.5	−0.308	−28.3
Cohort 5					**386**	343	301	262	1292	27.5	14	1.2	−0.283	−27.5
Cohort 6						**714**	637	566	1917	27.6	9	0.8	−0.266	−27.6
**Total**	**568**	**890**	**1515**	**2047**	**2219**	**2694**	**2389**	**2088**	**14,410**					

*Note:* The total number of children enrolled across all six cohorts is **3693**.

a Estimated by dividing the crude attrition by the length of the follow up period in years (e.g. 2.8 years for Cohort 1).

**Table 2:** Alternative measures of household welfare used in the study.

Poverty measure	Poverty dimension	Definition
Monthly household expenditures	Money-metric indicator of poverty	Computed by dividing the monthly total household consumption expenditure by the household size, considering a child to be equivalent of half an adult.
Asset index	Long term wealth	Derived from ownership of different assets both within the household and at other locations (radio, TV, car, motorcycle, stove, refrigerator, and phone) using principal component analysis (PCA).
Food security Index	A proxy for availability of food	Constructed from the frequency of buying staple food, number of meals served in the last two days, frequency of luxury foods and number of days the family slept without eating, using PCA
Subjective poverty	Relative poverty	Constructed from a 10-scale perceived level of poverty (1 for very poor and 10 for very rich).

**Table 3a:** Correlation between four alternative measures of poverty.

	Expenditures poverty	Assets poverty	Food poverty	Subjective poverty
Expenditures poverty	1.000			
Assets poverty	0.175 (0.000)	1.000		
Food poverty	0.312 (0.000)	0.246 (0.000)	1.000	
Subjective poverty	0.072 (0.000)	0.168 (0.000)	0.260 (0.000)	1.000

**Table 3b:** Sample characteristics – poverty measures.

	Child level (at enrollment)		Full sample (observations)	
	*N*	%	*N*	%
Expenditures poverty				
Poorest	1,340	36.3	4,773	33.1
Middle	1,256	34.0	4,769	33.1
Least poor	1,096	29.7	4,768	33.1
Missing^a^	1	0.0	100	0.7
Assets poverty				
Poorest	1,267	34.3	4,786	33.2
Middle	1,289	34.9	4,754	33.0
Least poor	1,136	30.8	4,770	33.1
Missing^a^	1	0.0	100	0.7
Food poverty				
Poorest	1,145	31.0	4,770	33.1
Middle	1,288	34.9	4,770	33.1
Least poor	1,259	34.1	4,770	33.1
Missing^a^	1	0.0	100	0.7
Subjective poverty				
Poorest (rank 1–3)	1,410	38.2	5,509	38.2
Middle (rank 4–5)	1,178	31.9	4,622	32.1
Least poor (rank 6–10)	1,104	29.9	4,179	29.0
Missing^a^	1	0.0	100	0.7
**Total**	3,693		14,410	

a Missing values are exclused from the bivariate and multivariate analyses.

**Table 3c:** Sample characteristics – control variables.

	Child level (at enrollment)		Full sample (observations)	
	*N*	%	*N*	%
Child age (in months)				
<6	3,062	82.9	3,291	22.8
6–11	631	17.1	2,945	20.4
12–23			5,548	38.5
24+			2,626	18.2
Child sex				
Male	1,856	50.3	7,288	50.6
Female	1,837	49.7	7,122	49.4
Mother's education (ref: No.)				
Education/primary incomplete	1104	29.9	4572	31.7
Primary complete	1718	46.5	6464	44.9
Secondary +	871	23.6	3374	23.4
Mother's length of residence in the study area				
0–2 years	1658	44.9	4802	33.3
3–5 years	752	20.4	3514	24.4
6+ years	1283	34.7	6094	42.3
Mother's marital status				
Not in union	555	15.0	2959	20.5
In a union	3138	85.0	11,451	79.5
Mother's parity				
1	1217	33.0	4508	31.3
2	1031	27.9	4066	28.2
3+	1445	39.1	5836	40.5
Household size				
<4	1484	40.2	5082	35.3
4–5	1534	41.5	6301	43.7
6+	675	18.3	3027	21.0
Household environment				
Poorest	1421	38.5	4778	33.2
Middle	1282	34.7	5504	38.2
Least poor	989	26.8	4028	28.0
Missing^a^	1	0.0	100	0.7
Slum residence				
Korogocho	1886	51.1	7625	52.9
Viwandani	1807	48.9	6785	47.1
**Total**	3693		14,410	

a Missing values are exclused from the bivariate and multivariate analyses.

**Table 4:** Bivariate analysis of the effects of household poverty status on child stunting, stratified by age.

	Poverty measure			
	Model with expenditures poverty	Model with assets poverty	Model with food poverty	Model with subjective poverty
Overall sample				
Poorest	0.83⁎⁎	1.26⁎⁎	1.40⁎⁎⁎	1.30⁎⁎⁎
Middle	0.84⁎	0.99	1.24⁎⁎	1.22⁎⁎
Least poor	1.00	1.00	1.00	1.00
				
Among <6				
Poorest	1.18	1.23	1.11	1.16
Middle	1.00	1.49⁎	1.18	1.23
Least poor	1.00	1.00	1.00	1.00
				
Among 6–11				
Poorest	1.21	1.68⁎	2.29⁎⁎⁎	1.13
Middle	0.93	0.95	1.64⁎	1.05
Least poor	1.00	1.00	1.00	1.00
				
Among 12–23				
Poorest	1.67⁎⁎	1.87⁎⁎⁎	1.93⁎⁎⁎	1.63⁎⁎
Middle	1.60⁎⁎	1.13	1.62⁎⁎	1.49⁎
Least poor	1.00	1.00	1.00	1.00

Among 24+				
Poorest	3.12⁎	8.05⁎⁎⁎	3.83†	12.39⁎⁎⁎
Middle	2.26⁎	1.84	1.80^†^	2.39
Least poor	1.00	1.00	1.00	1.00

† *p*<.10.

⁎ *p*<.05.

⁎⁎ *p*<.01.

⁎⁎⁎ *p*<.001

**Table 5:** Multivariate analysis^a^ of the effects of household poverty status on child stunting, stratified by age.

		Poverty measure			
		Model with expenditures poverty	Model with assets poverty	Model with food poverty	Model with subjective poverty
Overall sample					
Poorest		1.07	1.38⁎	1.13	1.24⁎
Middle		1.03	1.03	1.21†	1.18†
Least poor		1.00	1.00	1.00	1.00

Among <6					
Poorest		1.30	1.28	1.19	1.26
Middle		1.09	1.42†	1.19	1.27
Least poor		1.00	1.00	1.00	1.00

Among 6–11					
Poorest		1.10	1.39	2.16⁎⁎	1.23
Middle		0.94	0.92	1.68⁎	1.15
Least poor		1.00	1.00	1.00	1.00

Among 12–23					
Poorest		1.23	1.31	1.19	1.44⁎
Middle		1.17	1.04	1.26	1.39⁎
Least poor		1.00	1.00	1.00	1.00

Among 24+					
Poorest		1.33	3.91⁎⁎	1.27	4.35⁎⁎
Middle		1.54	1.65	1.10	2.58⁎
Least poor		1.00	1.00	1.00	1.00

^***^p<.001.

a All analyses include all control variables as in Table 6.

† *p*<.10.

⁎ *p*<.05.

⁎⁎ *p*<.01.

**Table 6:** Multivariate analysis of the determinants of child stunting.

	Poverty measure			
	Model with expenditures poverty	Model with assets poverty	Model with food poverty	Model with subjective poverty
Poverty status (ref: least poor) (**as reported in**Table 5**)**				
Poorest	1.07	1.38⁎	1.13	1.24⁎
Middle	1.03	1.03	1.21†	1.18†
Least poor	1.00	1.00	1.00	1.00
Child age (ref: <6 months)				
<6	0.14⁎⁎⁎	0.14⁎⁎⁎	0.14⁎⁎⁎	0.14⁎⁎⁎
6–11	1.00	1.00	1.00	1.00
12–23	8.53⁎⁎⁎	8.51⁎⁎⁎	8.52⁎⁎⁎	8.47⁎⁎⁎
24+	11.90⁎⁎⁎	11.81⁎⁎⁎	11.93⁎⁎⁎	11.79⁎⁎⁎
Child sex (ref: male)				
Male	1.00	1.00	1.00	1.00
Female	0.40⁎⁎⁎	0.41⁎⁎⁎	0.40⁎⁎⁎	0.40⁎⁎⁎
Mother's education (ref: none/primary incomplete)				
None/primary incomp	1.00	1.00	1.00	1.00
Primary complete	0.86	0.87	0.85	0.86
Secondary +	0.56⁎⁎⁎	0.56⁎⁎	0.55⁎⁎⁎	0.56⁎⁎⁎
Mother's length of residence in the study area (ref: 0–2 years)				
0–2 years	1.00	1.00	1.00	1.00
3–5 years	0.85	0.85	0.85	0.85
6+ years	0.83	0.83	0.84	0.85
Mother's marital status (ref: not in union)				
Not in union	1.00	1.00	1.00	1.00
In a union	0.65⁎⁎⁎	0.67⁎⁎⁎	0.65⁎⁎⁎	0.66⁎⁎⁎
Mother's parity (ref: 1)				
1	1.00	1.00	1.00	1.00
2	1.45⁎	1.45⁎	1.46⁎	1.44⁎
3+	1.54⁎⁎	1.54⁎⁎	1.55⁎⁎	1.52⁎⁎
Household size (ref: <4)				
<4	1.00	1.00	1.00	1.00
4–5	0.93	0.94	0.93	0.95
6+	0.96	0.99	0.98	0.99
Household environment				
Poorest	0.83	0.81	0.83	0.83
Middle	1.09	1.09	1.08	1.11
Least poor	1.00	1.00	1.00	1.00
Slum residence (ref: Korogocho)				
Korogocho	1.00	1.00	1.00	1.00
Viwandani	0.81†	0.88	0.80†	0.83

† *p*<.10.

⁎ *p*<.05.

⁎⁎ *p*<.01.

⁎⁎⁎ *p*<.001.

## Appendix 1

(See Table A1).

## Appendix 2

(See Table A2).
